# *Nax* loci affect SOS1-like Na^+^/H^+^ exchanger expression and activity in wheat

**DOI:** 10.1093/jxb/erv493

**Published:** 2015-11-19

**Authors:** Min Zhu, Lana Shabala, Tracey A Cuin, Xin Huang, Meixue Zhou, Rana Munns, Sergey Shabala

**Affiliations:** ^1^School of Land and Food and Tasmanian Institute for Agriculture, University of Tasmania, Private Bag 54, Hobart, Tas 7001, Australia; ^2^Institute for Molecular Plant Physiology and Biophysics, Julius-von-Sachs Platz 2, D-97082 Würzburg, Germany; ^3^School of Plant Biology and ARC Centre of Excellence in Plant Energy Biology, The University of Western Australia, Crawley WA 6009, Australia; ^4^CSIRO Agriculture, Canberra, ACT 2601, Australia

**Keywords:** HKT transporter, potassium, salinity stress, sequestration, sodium, xylem loading.

## Abstract

Salinity stress tolerance in durum wheat is strongly associated with a plant’s ability to control Na^+^ delivery to the shoot. Two loci, termed *Nax1* and *Nax2*, were recently identified as being critical for this process and the sodium transporters *HKT1;4* and *HKT1;5* were identified as the respective candidate genes. These transporters retrieve Na^+^ from the xylem, thus limiting the rates of Na^+^ transport from the root to the shoot. In this work, we show that the *Nax* loci also affect activity and expression levels of the SOS1-like Na^+^/H^+^ exchanger in both root cortical and stelar tissues. Net Na^+^ efflux measured in isolated steles from salt-treated plants, using the non-invasive ion flux measuring MIFE technique, decreased in the sequence: Tamaroi (parental line)>*Nax1*=*Nax2*>*Nax1:Nax2* lines. This efflux was sensitive to amiloride (a known inhibitor of the Na^+^/H^+^ exchanger) and was mirrored by net H^+^ flux changes. *TdSOS1* relative transcript levels were 6–10-fold lower in *Nax* lines compared with Tamaroi. Thus, it appears that *Nax* loci confer two highly complementary mechanisms, both of which contribute towards reducing the xylem Na^+^ content. One enhances the retrieval of Na^+^ back into the root stele via HKT1;4 or HKT1;5, whilst the other reduces the rate of Na^+^ loading into the xylem via SOS1. It is suggested that such duality plays an important adaptive role with greater versatility for responding to a changing environment and controlling Na^+^ delivery to the shoot.

## Highlight

Improved salinity stress tolerance in wheat *Nax* lines is achieved by transcriptional and post-translational down-regulation of Na^+^ loading into the xylem by a SOS1 transporter.

## Introduction

Soil salinity severely affects plant growth and limits agricultural crop production (Qadir *et al.*, 2014; Shabala *et al.*, 2014). Approximately 20% of the world’s cultivated land, which accounts for over 6% of the world total area, is currently threatened by salinity (Rengasamy, 2010). An elevated salt concentration in the soil leads to the accumulation of toxic concentrations of Na^+^ in the leaves (Munns and Tester, 2008; Rahnama *et al.*, 2010). Consequently, control of Na^+^ long-distance transport and the ability to retrieve Na^+^ from the xylem are considered amongst the most essential traits conferring salinity tolerance (Munns and Tester, 2008; Shabala *et al.*, 2013).

Wheat is one of the most important cereal crops worldwide, providing approximately one-fifth of the total calorific input of the world’s population (Shewry, 2009). Among wheat species, durum (pasta) wheat (*Triticum turgidum* ssp. *durum*) is generally less tolerant to salt stress than bread wheat (*Triticum aestivum*) (Cuin *et al.*, 2009), mainly due to the high rates of Na^+^ accumulation and poor K^+^/Na^+^ discrimination (Gorham *et al.*, 1990; Munns and James, 2003).

Two loci that reduced Na^+^ accumulation in the shoot, named *Nax1* and *Nax2*, were discovered in an unusual durum wheat (James *et al.*, 2006). These loci had been transferred there from the ancestral wheat species einkorn (*Triticum monococcum*) in order to introduce rust resistance genes into modern wheat (James *et al.*, 2006). These loci are not present in modern durum or bread wheat, so they were crossed into the current durum cultivar Tamaroi, and near-isogenic lines were developed containing either the *Nax1* or *Nax2* loci or both. These lines had lower rates of Na^+^ transport from the roots to the shoots, the result of a lower rate of net Na^+^ loading into the xylem (James *et al.*, 2006). Both loci could unload Na^+^ from the xylem in the root, while *Nax1* could also unload Na^+^ from the xylem at the leaf base (the sheath) so leading to a high Na^+^ ratio between the sheath and the blade (James *et al.*, 2006). *Nax1* and *Nax2* lines also had higher rates of K^+^ transport from the root to the shoot, resulting in an enhanced discrimination of K^+^ over Na^+^ (James *et al.*, 2006). Using fine-mapping, the candidate gene for *Nax1* was identified on chromosome 2A as a Na^+^ transporter of the *HKT* gene family *TmHKT1;4* (Huang *et al.*, 2006). The candidate gene for *Nax2* was localized on chromosome 5A and identified as *TmHKT1;5* (Byrt *et al.*, 2007). It was localized on the plasma membrane of cells surrounding the xylem and, when crossed into an elite Australian durum cultivar, was found to confer a yield benefit of 25% on saline soil in a farmer’s field (Munns *et al.*, 2012). A recent study found that a closely related gene in bread wheat, *TaHKT1;5-D,* is also localized on the plasma membrane in the root stele and operates in retrieving Na^+^ from the xylem vessels thus restricting the transport of Na^+^ from the root to the leaves in bread wheat (Byrt *et al.*, 2014).

While Na^+^ retrieval from the xylem before it reaches the sensitive photosynthetic tissues is indeed essential for plant performance under saline conditions, it is also important to reduce the amounts of Na^+^ initially loaded into the xylem. It was argued (Shabala *et al.*, 2010, 2013) that the ideal scenario for a plant would be quickly to send the amount of Na^+^ to the shoot that is required in order to achieve full osmotic adjustment rapidly and to maintain a normal growth rate (hence, no yield penalties). Once this is achieved, it would be advantageous for a plant to reduce the rate of xylem Na^+^ loading to the absolute minimum for maintaining cell turgor in growing tissues while, at the same time, preventing excessive Na^+^ from being accumulated in photosynthetically-active and fully-grown leaf tissues. This poses a question: what is the molecular nature of the mechanisms mediating xylem Na^+^ loading and the modes of their control? Recent thermodynamic analysis has suggested that channel-mediated xylem Na^+^ loading dominates at the early stages of salt stress (minutes to hours), while longer exposure to salinity (hours and days) will require thermodynamically-active xylem Na^+^ loading (Shabala, 2013). Two possible candidates were proposed, one a SOS1 Na^+^/H^+^ exchanger (Shi *et al.*, 2002) and another, a cation–Cl (CCC) co-transporter (Colmenero-Flores *et al.*, 1999).

The salt-overly-sensitive (SOS) signal transduction pathway is regarded as a key mechanism for maintaining intracellular ion homeostasis under saline conditions (Zhu *et al.*, 1998; Hasegawa *et al.*, 2000; Sanders *et al.*, 2000; Zhu, 2001). According to the current view, elevated Na^+^ causes increases in cytosolic free Ca^2+^ (Knight *et al.*, 1997), which can be sensed by SOS3, a myristoylated Ca^2+^-binding protein (Liu and Zhu, 1998; Ishitani *et al.*, 2000). SOS3 interacts with and activates SOS2 (a serine/threonine protein kinase), thus forming a SOS2/SOS3 complex (Halfter *et al.*, 2000; Liu *et al.*, 2000). *AtSOS1* is identified as a Na^+^/H^+^ antiporter, localized in epidermal cells at the root tip and also in parenchyma cells at the xylem/symplast boundary of roots, stems, and leaves where it controls long-distance Na^+^ transport (Shi *et al.*, 2002). Overexpression of *SOS1* in transgenic *Arabidopsis* has been shown to improve salt tolerance (Shi *et al.*, 2003). Furthermore, Feki *et al.* identified the *TdSOS1* orthologue from durum wheat. The heterologous expression of *TdSOS1* in a yeast strain lacking endogenous Na^+^ efflux proteins showed complementation involving cation efflux (Feki *et al.*, 2011). Importantly, expression of a truncated form of wheat *TdSOS1* in the *Arabidopsis sos1-1* mutant exhibited improved salt tolerance (Feki *et al.*, 2014).

In this study, we used *Nax1* and *Nax2* durum wheat lines to provide supporting evidence of a role for SOS1-mediated Na^+^ loading into the xylem in these species. We tested the hypothesis that reduced Na^+^ accumulation in the shoot of *Nax* lines could be conferred not only by higher Na^+^ retrieval from the xylem, but also by reduced Na^+^ loading into the xylem. Our electrophysiological and molecular data fully support this hypothesis and suggest that the *Nax* loci regulate activity and expression levels of a SOS1-like Na^+^/H^+^ exchanger in the xylem tissue of wheat and that down-regulation of this transporter in *Nax* lines improves plant performance under saline conditions. This mechanism operates in addition to, or instead of, the reported increased Na^+^ retrieval from the xylem by the HKT transporter. This reduces the overall net xylem Na^+^ loading and accumulation in the shoot, thus increasing salinity tolerance.

## Materials and methods

### Plant material and growth conditions

Durum wheat (*Triticum turgidum* L. ssp. *durum* Desf.) seeds of cv. Tamaroi and BC_5_F_2_ *Nax* lines were a kind gift from Dr Richard James (CSIRO Plant Industry, Canberra). Tamaroi, like all durum and bread wheat cultivars, lacks the *Nax* loci which originated from the diploid ancestral wheat, *Triticum monococcum* (James *et al.*, 2006). These loci were back-crossed into the current durum cultivar Tamaroi, and near-isogenic BC_5_F_2_ lines were developed using specific molecular markers (James *et al.*, 2006). *Nax* lines were homozygous for *Nax1*, *Nax2*, or both *Nax1* and *Nax2*. The candidate gene for *Nax1* is *TmHKT1;4-A2* on chromosome 2A (Huang *et al.*, 2006) and for *Nax2* is *TmHKT1;5-A* on chromosome 5A (Byrt *et al.*, 2007). *SOS1* is located on chromosome 3 (Mullan *et al.*, 2007), and there was no selection for this gene during back-crossing, so all lines have the same *SOS1* gene allele as the parental cultivar Tamaroi.

Seeds were surface-sterilized with 10% bleach (King White, Victoria, Australia) for 10min, rinsed thoroughly with deionized water, then grown hydroponically for 6 d in the dark in an aerated Basic Salt Medium (BSM) containing 0.1mM CaCl_2_ and 0.5mM KCl (pH=5.6; non-buffered).

### Non-invasive ion flux measurements

Net ion fluxes were measured using non-invasive ion-selective vibrating microelectrodes (the MIFE technique; University of Tasmania, Hobart, Australia). The principles of MIFE ion flux measurements are described in full elsewhere (Shabala *et al.*, 1997) and all the details of microelectrode fabrication and calibration are available in our previous publications (Shabala and Shabala, 2002; Shabala *et al.*, 2006). Liquid ionic exchangers used in this work are the commercially available ionophore cocktails (60031 for K^+^; 71176 for Na^+^; 95297 for H^+^; all from Fluka, Busch, Switzerland).

### Root K^+^ and H^+^ flux measurements

One hour prior to measurement, 6-d-old wheat seedlings were immobilized, with their roots placed horizontally in a 10ml Perspex measuring chamber containing the bathing medium as described elsewhere (Chen *et al.*, 2005; Bose *et al.*, 2014). Two ion-selective microelectrodes, one for K^+^, the other for H^+^, were used simultaneously, with the electrode tips aligned and positioned 50 μm above the root surface. Once steady-state fluxes were reached (40–60min after immobilization), measurements commenced. Net fluxes of ions were measured for 5–10min from the mature (~15–20mm from the tip) root zone. The salinity treatment (80mM NaCl) was then administered and net K^+^ and H^+^ fluxes measured for a further 60min.

### Root Na^+^ flux measurements

A so-called ‘recovery protocol’ (Cuin *et al.*, 2011) was used to quantify the activity of a Na^+^ efflux system in epidermal and stelar root tissues. Six-day-old wheat seedlings were treated with 150mM NaCl for a further 24h in the dark. A seedling was then transferred to a 10ml Perspex measuring chamber containing the bathing medium and 150mM NaCl. After 1h adaption, roots were quickly but thoroughly rinsed with a 10mM CaCl_2_ solution to remove surface and apoplastic NaCl before being transferred to a clean chamber containing Na^+^-free BSM solution. Net ion fluxes were measured for approximately 60min from either the mature root epidermis of the seminal root (~15–20mm from the tip), or root elongation zone. The first 2min of recording were ignored during analysis to eliminate any confounding effect of the Donnan exchange in the cell wall (see Cuin *et al.*, 2011, for justification and details).

For measurements from the xylem parenchyma, the root stele was mechanically isolated as described earlier (Shabala *et al.*, 2010). An apical stelar segment was cut (the first 5–7mm of the stele), immobilized in a Perspex chamber in BSM and left to recover for 4–6h in the presence of 50mM NaCl. The recovery protocol (see above) was then applied to quantify the activity of a Na^+^ efflux system at the xylem parenchyma interface.

### RNA extraction and RT-qPCR experiments

Total RNA was extracted from ~100mg of roots from Tamaroi and *Nax* lines using the RNeasy Plant Mini Kit (Qiagen). First-strand cDNA was synthesized using the QuantiTect Reverse Tanscription Kit (Qiagen), which includes the genomic DNA removal step. Relative transcript levels were assayed by real-time PCR analysis using the Qiagen Rotor-gene Real-Time PCR system. The *TdSOS1* gene was amplified using two specific primers *TdSOS1*; 5′SOS (5′-ATTCCCTCAGGTGCTTCGTG-3′) and 3′SOS (5′-TTTCCTCGAGCAACCCAGTC-3′). The wheat actin gene (Genebank Accession No. AB181991.1) was used as an internal control for gene expression. The actin primers were AF (5′-TACACGAAGCGACATACAA-3′) and AR (5′-AATAGAGCCACCGATCCA-3′). RT-qPCR conditions were as follows: 95 °C for 5min, 94 °C for 30s (50 cycles), 58 °C for 30s, and 72 °C for 1min. Amplified products were detected using QuantiNova SYBR Green PCR Kit (Qiagen). Each data point represents six biological replicates in each sample, presented as the mean ±SE. The experiment was repeated three times, with consistent results.

### Statistical analysis

Data were analysed using one-way of variance, and treatment mean separations were performed using Duncan’s multiple range tests at the 5% level of significance in IBM Statistics 21.

## Results

### NaCl-induced ion flux response in root

**Fig. 1.**
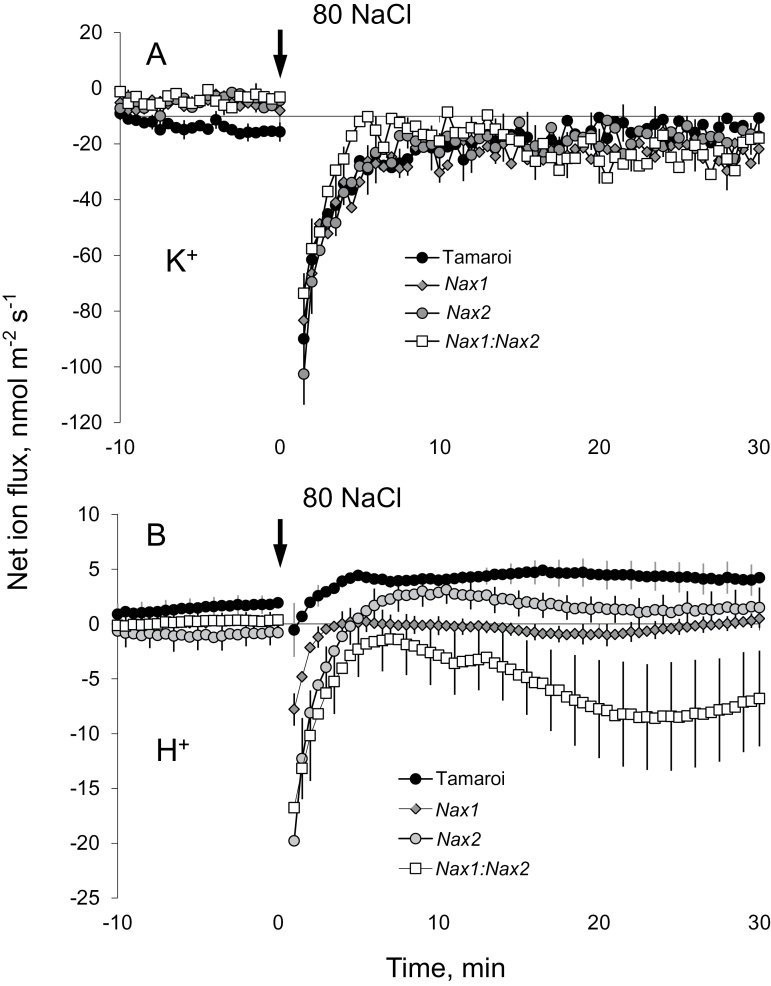
Transient K^+^ (A) and H^+^ (B) flux responses measured from the mature zone of the root epidermis from *Nax* lines and the parental Tamaroi (lacking *Nax* loci) in response to acute 80mM NaCl treatment. Mean ±SE (*n*=6). For all MIFE measurements, the sign convention is ‘efflux negative’.

The addition of 80mM NaCl caused significant changes in net ion (K^+^ and H^+^) fluxes from the mature root zone of the durum wheat cultivar Tamaroi, which lacks the *Nax* loci, as well as near-isogenic lines that contained the *Nax* loci; Fig. 1). Peak K^+^ efflux was reached within a few minutes of stress onset (Fig. 1A), followed by a gradual recovery of K^+^ flux (although it always remained negative, i.e. net efflux). No significant (*P* <0.05) differences in NaCl-induced K^+^ efflux kinetics were found between Tamaroi and any of the *Nax* lines. H^+^ fluxes measured in response to salt treatment were lower for the *Nax* lines compared with Tamaroi (Fig. 1B), with steady-state H^+^ flux values (measured 30min after stress onset) being Tamaroi>*Nax1*=*Nax 2*>*Nax1:Nax2* lines (Fig. 1B). Interestingly, an H^+^ efflux was observed in the *Nax1:Nax2* line, in contrast to the slight H^+^ influx found in Tamaroi and the other two *Nax* lines.

**Fig. 2.**
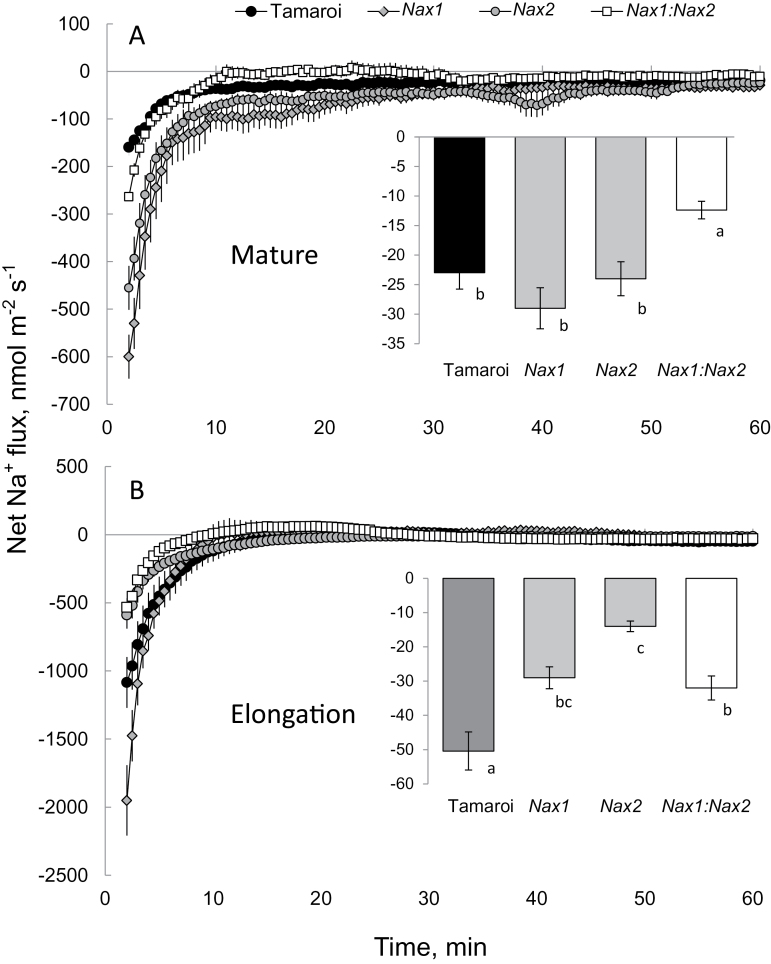
Net Na^+^ fluxes measured in ‘recovery protocols’ from (A) mature and (B) apical (elongation zone; ~2mm from root tip) zones of epidermal root cells of *Nax* lines and Tamaroi after transfer from 150mM NaCl solution (24h treatment) to sodium-free BSM. Mean ±SE (*n*=6). Inserts in each panel denote the steady-state Na^+^ efflux 60min after the removal of the salt treatment.

Transfer of salt-treated roots (150mM NaCl for 24h) to Na^+^-free solution resulted in a significant Na^+^ efflux from the root epidermis in both the mature and elongation zones (Fig. 2). Inserts in each panel denote steady-state Na^+^ efflux during the final 30min of the measurement. No clear trends emerged for the mature zone (see insert in Fig. 2A), but the Na^+^ efflux in the root elongation zone (where *SOS1* is predominantly expressed; Shi *et al.*, 2002) in all *Nax* lines was significantly lower than in Tamaroi (significant at *P* <0.05).

**Fig. 3.**
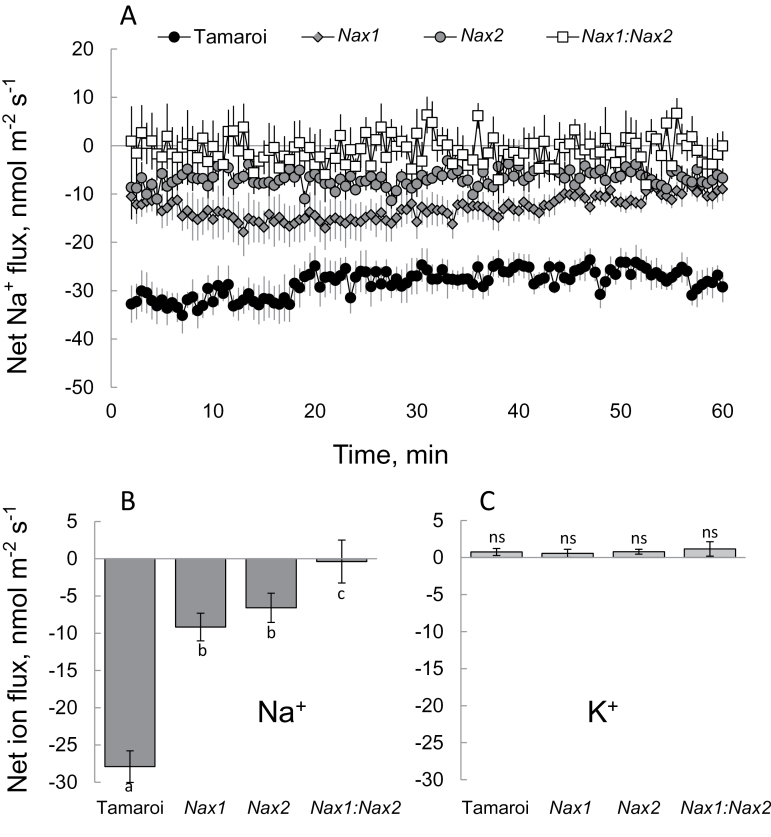
(A) Net Na^+^ fluxes measured in ‘recovery protocols’ from the root stele after 6h exposure to 50mM NaCl. (B, C) Mean Na^+^ (B) and K^+^(C) values measured from stelar tissue over the 60min interval after transferring the stele to a Na^+^-free solution. Mean ±SE (*n*=6).

To quantify the activity of the Na^+^ efflux system at the xylem parenchyma interface, the root stele was mechanically isolated as described in the Materials and Methods, and net ion fluxes were measured after transferring the stele from 50mM NaCl (pre-treated for 6h) to a Na^+^-free BSM. The net Na^+^ efflux in Tamaroi was significantly (*P* <0.05) higher than in the other three genotypes, while the *Nax1:Nax2* line had the lowest Na^+^ efflux. Net K^+^ fluxes were not significantly different from zero (Fig. 3C), indicating that the observed difference in measured Na^+^ flux was not an artefact originating from the possible poor discrimination of the Na^+^ microelectrode LIX between Na^+^ and K^+^ (see Chen *et al.*, 2005, for details).

### Ion flux profiles in leaf sheath and blade tissues

**Fig. 4.**
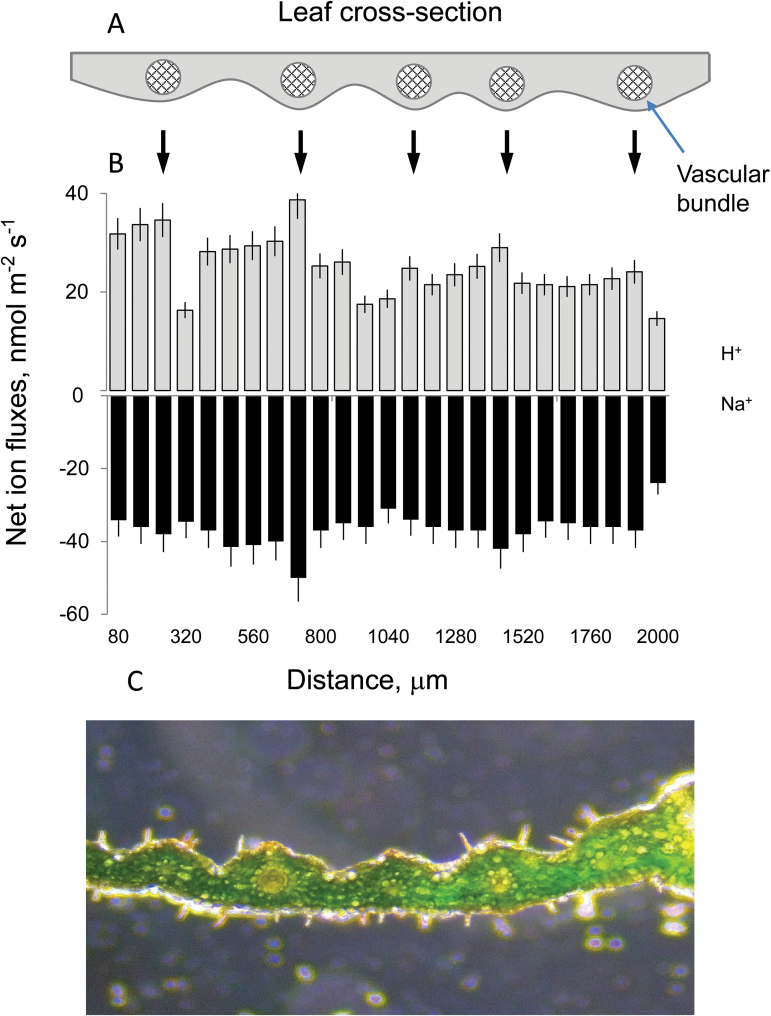
Ion flux profiles along the cross-section of wheat leaf. (A) Schematic model depicting the position of major veins in a wheat leaf. (B) Net H^+^ (grey bars) and Na^+^ (black bars) fluxes measured at different parts of the leaf (see panel A) from control plants to assess the leaf profile. Mean ±SE (*n*=4 individual leaves). (C) A microscopic image depicting a cross-section of a wheat leaf.

**Fig. 5.**
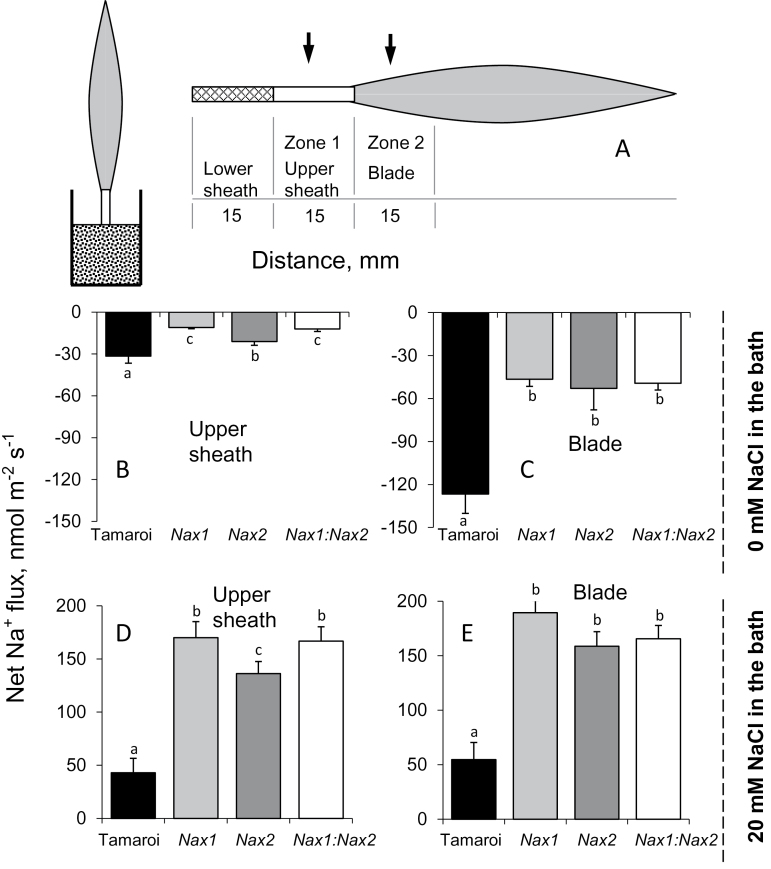
Activity of Na^+^ efflux systems in the leaf blade and upper sheath tissues of wheat lines. (A) A schematic diagram of the experimental protocol. Excised leaves were placed in a beaker containing 20mM NaCl solution with the sheath immersed in the solution to a depth of 15mm and treated for 2 d to accumulate salt. Particular attention was paid to ensure that the depth of insertion and the total surface area of the leaf exposed to salinity was the same in all treatments. (B, C) Steady-state Na^+^ fluxes measured from leaf segments isolated from the upper sheath (B, zone 1 in panel A) and leaf blade tissue (C, zone 2 in panel A) of leaves treated with 20mM NaCl as described above and then transferred into Na^+^-free BSM medium for MIFE measurements. (D, E) Net Na^+^ fluxes from the upper sheath (D) and leaf blade tissue (E) exposed to 20mM NaCl for 2 d, and measured in the presence of 20mM NaCl in the bathing medium. Mean ±SE (*n*=5).

Previous studies have pointed to the leaf sheath, as well as the roots, as the most likely locations of *HKT1;4* gene expression conferred by the *Nax1* loci (Huang *et al.*, 2006; James *et al.* 2006). Accordingly, the difference in net ion fluxes between the vascular bundles and leaf mesophyll in Tamaroi and *Nax* lines was compared. As the *HKT* genes involved in Na^+^ retrieval from the xylem are considered to be expressed in tissues surrounding the vascular bundle (Horie *et al.*, 2009), it was critical that the electrodes were positioned exactly above this tissue. To ensure this, methodological experiments were conducted, mapping cross-sectional ion flux profiles in wheat leaves (Fig. 4). Similar to our previous reports for bean mesophyll (Shabala *et al.*, 2002), the ion flux profiles in wheat leaves showed a strong correlation with leaf anatomy, with both the highest H^+^ influx and the highest Na^+^ efflux occurring from vascular bundles (Fig. 4B). Consequently, locations with the highest H^+^ influx and Na^+^ efflux activity were used to compare parental and *Nax* lines (depicted in Fig. 5).

When detached leaves were placed in 20mM NaCl, Tamaroi leaves showed significantly lower net Na^+^ uptake compared with all the *Nax* lines (a 3-fold difference; Fig. 5D, E; significant at *P* <0.05) in both the upper sheath and blade tissue. As the measured net flux is a sum of channel-mediated Na^+^ uptake and transporter-mediated Na^+^ efflux, one possible explanation for this observation may be higher Na^+^ leaf exclusion ability in Tamaroi. To check this, leaf segments were transferred to a Na^+^-free medium to reveal the specific contribution of Na^+^-efflux systems. Consistent with these data, the net Na^+^ efflux in the leaf blade tissue was much more pronounced in Tamaroi than in any of the *Nax* lines (Fig. 5B, C; significant at *P* <0.05). The same pattern was observed both in the upper sheath (Fig. 5D) and leaf blade (Fig. 5E) regions, although net Na^+^ efflux was about 4-fold stronger from the leaf blade.

### *Effect of* Nax *loci on* SOS1 *transcript level in roots*

**Fig. 6.**
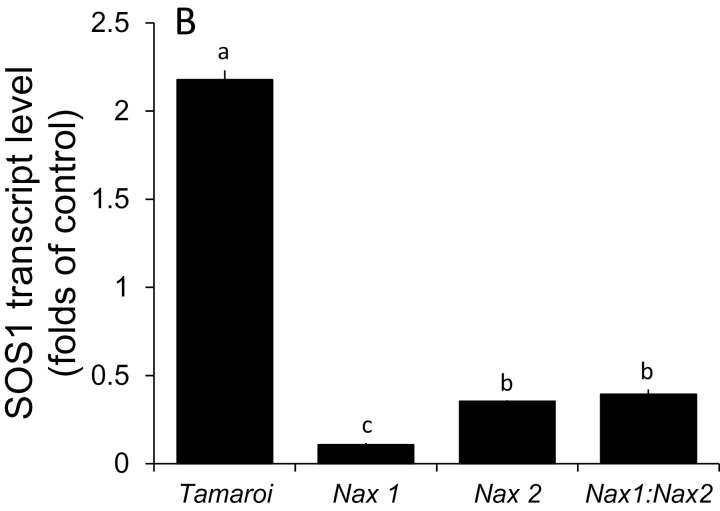
The relative transcript level of *TdSOS1* in roots of Tamaroi and *Nax* lines (6-d-old seedlings exposed to 150mM NaCl for 24h). Each data point represents six biological replicates in each sample, presented as mean ±SE.

The relative *SOS1* transcript level was up-regulated (compared with the control) in salt-treated (150mM for 3 weeks) Tamaroi roots (2.18-fold; Fig. 6), while it was down-regulated in all *Nax* lines (0.11-, 0.35-, and 0.39-fold for *Nax1*, *Nax2*, *Nax1:Nax2* lines, respectively, all significant at *P* <0.001).

## Discussion

The *Nax* loci restrict Na^+^ transport from the roots to the leaves. Their effect on net xylem loading of Na^+^ (James *et al.*, 2006), subsequent Na^+^ exclusion from leaves, and leaf longevity under saline conditions (James *et al.*, 2011), was, until now, presumed to be due entirely to the action of the Na^+^ transporters *HKT1:4* and *HKT1:5*, respectively contained within them (Huang *et al.*, 2006; Byrt *et al.*, 2007). We show here that the *Nax* loci also affect SOS1-like Na^+^/H^+^ exchanger expression and activity in durum wheat. While interpreting our data we assume a similarity in SOS1 function and expression pattern between *Arabidopsis* and wheat.

### *Activity of a SOS1-like Na* ^*+*^ */H* ^*+*^ *exchanger in root epidermis is suppressed in* Nax *lines*

As shown in Fig. 1B and Fig. 2B, net H^+^ influx and Na^+^ efflux in root epidermal cells in *Nax* lines were much lower than those in Tamaroi. These observations are fully consistent with the notion that the *Nax* loci also affect SOS1-like Na^+^/H^+^ exchanger activity in the root epidermis. First, the observed patterns were only found at the root apex (where SOS1 transporters are most strongly expressed; Shi *et al.*, 2002) but not in the mature root zone (Fig. 2). Second, the measured Na^+^ flux was sensitive to amiloride, a known inhibitor of the Na^+^/H^+^ exchanger (data not shown; but see Cuin *et al.*, 2011, for supporting evidence). In contrast to animals, higher plants lack ATP-driven Na^+^-pumps, so rely on Na^+^/H^+^ exchangers to efflux Na^+^ back to the apoplast. It is estimated that a typical glycophyte plant effluxes about 90% of the Na^+^ that enters a root cell (Davenport *et al*., 2005); the operation of a SOS1 plasma membrane Na^+^/H^+^ exchanger seems to be essential to achieve this goal (Shi *et al.*, 2002, 2003). Overexpression of *AtSOS1* has been shown to improve salt tolerance in transgenic *Arabidopsis* (Shi *et al.*, 2003). In addition, *OsSOS1* has been characterized in rice and it demonstrates a capacity for Na^+^/H^+^ exchange in plasma membrane vesicles of yeast (*Saccharomyces cerevisiae*) cells, reducing their net cellular Na^+^ content (Martinez-Atienza *et al.*, 2007). When the activity of the SOS1 exchanger is suppressed under saline conditions, Na^+^ exclusion and H^+^ uptake in the root epidermis is reduced. This is what is observed here for all *Nax* lines.

### *The SOS1-like exchanger plays a substantial role in xylem Na* ^*+*^ *loading in wheat and its activity is reduced in stelar tissues of* Nax *lines*

In *Arabidopsis*, *SOS1* genes are preferentially expressed in stelar root tissues (Shi *et al.*, 2002) and are considered to function in xylem Na^+^ loading (Shi *et al.*, 2002). Our finding that net Na^+^ efflux is significantly higher in Tamaroi compared with any *Nax* lines (Fig. 3) is consistent with this proposal and also provides strong supportive evidence for the inhibition of a SOS1-like exchanger by the *Nax* loci. Qualitatively similar patterns were observed in both the upper sheath (Fig. 5B) and blade of the leaf (Fig. 5C). Inevitably, a certain amount of Na^+^ will penetrate ‘the first line of defence’ (Na^+^ exclusion from the root epidermis) and enter the xylem. Here, it will either be (i) retrieved back into the stele by HKT transporters or (ii) transported to the shoot to be compartmentalized by leaf vacuoles where it can contribute to osmotic adjustment. Tamaroi was observed to have significantly more xylem Na^+^ loading than any of the *Nax* lines, due to the normal function of SOS1. Thus, it appears that the *Nax* loci confer two highly complementary mechanisms: an enhanced retrieval of Na^+^ back into the root stele (as reported elsewhere: Blumwald *et al.*, 2000; Shi *et al.*, 2002), and a reduced rate of Na^+^ loading into the xylem in the first instance (reported here). Both contribute to the same aim: reducing the xylem Na^+^ content. It can be speculated that such duality plays an important adaptive role and provides more flexibility to plants. Indeed, as shown in this work, the *Nax* loci may suppress the activity of a SOS1-like Na^+^/H^+^ exchanger in both epidermal (Fig. 2) and stelar (Fig. 3) tissues. This suppression reduces a plant’s ability to exclude Na^+^ from uptake but, at the same time, the rate of Na^+^ entering the xylem is also reduced. This should result in more Na^+^ staying in the roots, to be used either for osmotic adjustment (Shabala and Lew, 2002), or salt stress-signalling (Wu *et al.*, 2015) purposes. Also, from general point of view, it may be beneficial for plants to have another ‘back-up’ mechanism when challenged by salinity stress, in case one of the systems fails to operate.

The reduced SOS1-like activity in the *Nax* lines could be explained (at least partially) by the reduced level of the *SOS1*-transcript, as revealed by RT-qPCR experiments (Fig. 6). The down-regulation of *TdSOS1* in the root of the *Nax* line under saline condition could explain why the function of the SOS1 exchanger was altered in both the root epidermis and stele compared with the parental line Tamaroi. It could be speculated that the *Nax* loci, which consist of a short chromosome segment originating from *Triticum monococcum* as well as the HKT genes, contain some ‘regulating genes’, which have a negative feedback on the *TdSOS1* expression at the transcription level. This issue will be addressed in a future study.

### Root ion homeostasis under saline conditions: an improved model

**Fig. 7.**
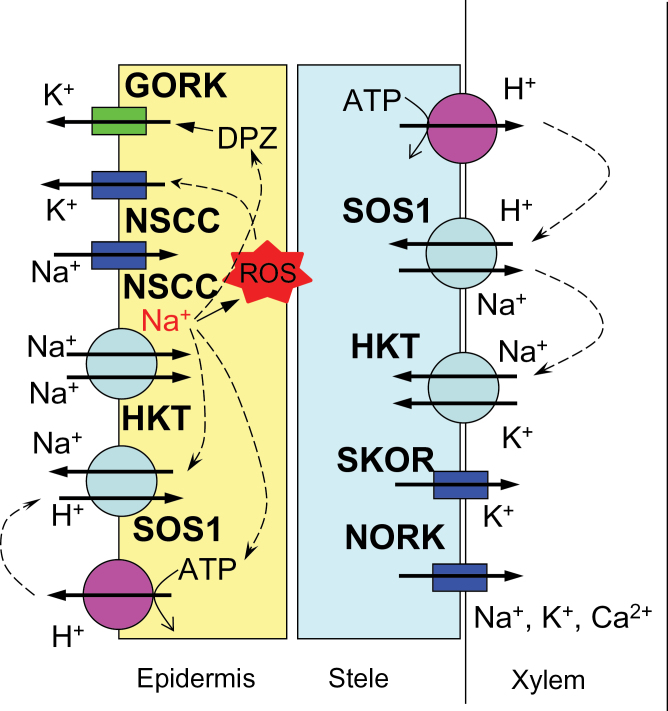
A model depicting the mechanisms contributing to root ion homeostasis under saline conditions. See the text for details.

Based on our reported results, the following model can be suggested (Fig. 7). Na^+^ enters the cell via non-selective cation channels (NSCC, Demidchik and Maathuis, 2007) and/or HKT transporters (Laurie *et al.*, 2002; Garciadeblas *et al.*, 2003; Haro *et al.*, 2005; Horie *et al.*, 2001, 2007), depolarizing the plasma membrane and resulting in a substantial K^+^ leak from the root epidermis (Fig. 1), mediated by GORK channels (Anschutz *et al.*, 2014; Pottosin and Shabala, 2014). Increased cytosolic Na^+^ can lead to the accumulation of ROS (Vass *et al.*, 1992; Allakhverdiev *et al.*, 2002), further exacerbating K^+^ efflux from cytosol via ROS-activated K^+^-permeable NSCC (Pottosin and Shabala, 2014). No difference in any of above mechanisms exists between Tamaroi and the *Nax* lines. The major bulk of Na^+^ will be excluded by plasma membrane-located SOS1 Na^+^/H^+^ exchangers, fuelled by the H^+^-ATPase. *Nax* loci suppress (directly or indirectly) the transcript level of the SOS1 gene (Fig. 6) and its activity (Fig. 2). Some of the Na^+^ accumulated in the root cortex is loaded into the xylem, mediated by both passive (at the early stages of salt stress; Shabala *et al.*, 2013) and active (SOS1-mediated) transport systems. In *Nax* lines, the rate of Na^+^ loading is suppressed, at either the transcriptional or functional level (or both). Some of the loaded Na^+^ is removed by HKT transporters located at the xylem parenchyma interface (Munns and Tester, 2008; Horie *et al.*, 2009); an ability much more pronounced in the *Nax* lines. Hence, xylem Na^+^ loading is controlled by two highly complementary uptake and release systems, providing the plant with a greater versatility to respond to a changing environment and to control Na^+^ delivery to the shoot.
